# Flexible piezoelectric materials and strain sensors for wearable electronics and artificial intelligence applications

**DOI:** 10.1039/d4sc05166a

**Published:** 2024-09-27

**Authors:** Yanyu Chen, Xiaohong Zhang, Chao Lu

**Affiliations:** a College of Chemistry, Chemical Engineering and Materials Science, Soochow University Suzhou Jiangsu 215123 China chaolu@suda.edu.cn; b Institute of Functional Nano & Soft Materials, Soochow University Suzhou Jiangsu 215123 China

## Abstract

With the rapid development of artificial intelligence, the applications of flexible piezoelectric sensors in health monitoring and human–machine interaction have attracted increasing attention. Recent advances in flexible materials and fabrication technologies have promoted practical applications of wearable devices, enabling their assembly in various forms such as ultra-thin films, electronic skins and electronic tattoos. These piezoelectric sensors meet the requirements of high integration, miniaturization and low power consumption, while simultaneously maintaining their unique sensing performance advantages. This review provides a comprehensive overview of cutting-edge research studies on enhanced wearable piezoelectric sensors. Promising piezoelectric polymer materials are highlighted, including polyvinylidene fluoride and conductive hydrogels. Material engineering strategies for improving sensitivity, cycle life, biocompatibility, and processability are summarized and discussed focusing on filler doping, fabrication techniques optimization, and microstructure engineering. Additionally, this review presents representative application cases of smart piezoelectric sensors in health monitoring and human–machine interaction. Finally, critical challenges and promising principles concerning advanced manufacture, biological safety and function integration are discussed to shed light on future directions in the field of piezoelectrics.

## Abstract 2

This review covers the recent advances in flexible piezoelectric materials, which show great potential for strain sensors in wearable electronics and artificial intelligence, focusing on microstructure engineering and fabrication techniques.

## Graphical abstract

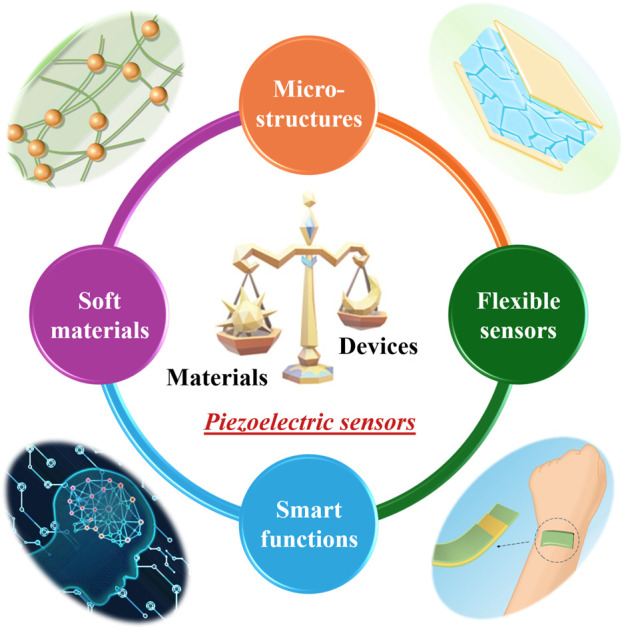

## 1 Introduction

In recent years, with the rapid development of advanced technologies such as the Internet of Things (IoT) and artificial intelligence,^1^ wearable electronic devices have attracted much attention due to their wide applications in biomedical,^2–4^ environmental monitoring,^5–7^ energy harvesting^8–10^ and other fields.^11,12^ As a key component, flexible strain sensors play a fundamental role in converting external physical information into electrical signals. The strain sensors can be classified into four primary categories according to the sensing mechanisms: piezoresistive,^13,14^ capacitive,^15,16^ triboelectric^17,18^ and piezoelectric mechanisms.^19,20^ Advanced wearable sensors require high integration, miniaturization and low power consumption, which cannot be met by piezoresistive and capacitive sensors with external power supply and limited forms. Compared with triboelectric sensors, piezoelectric sensors have the advantages of superior power density and high energy conversion efficiency,^21^ making them a promising option for self-powered, portable and wearable electronics. Piezoelectric sensors also exhibit characteristics such as an ultrathin structure, high sensitivity, fast response, stable output and less susceptibility to external electromagnetic signals, which have attracted considerable attention in recent years.

Piezoelectric materials can transduce applied stress into electrical signals depending on the piezoelectric effect.^22^ The piezoelectric effect refers to the phenomenon where applied mechanical stress induces an electric polarization and hence an electric potential across the material, which is directly proportional to the magnitude of mechanical stress. Traditional piezoelectric materials such as lead zirconate titanate (PZT), barium titanate (BaTiO_3_) and zinc oxide (ZnO) have excellent piezoelectric properties, but their high hardness and low tenacity greatly limit their applications in flexible sensors. The safety hazards of lead containing materials also cannot be ignored, as they have toxic effects on the environment and organisms.^23^ Piezoelectric polymer materials offer several benefits over traditional ones, including biocompatibility, flexibility and a high level of processability.^24^ Among the various polymer materials exhibiting piezoelectricity, PVDF, P(VDF-TrFE) and hydrogels are more commonly considered due to their excellent physical and chemical properties. The piezoelectric coefficients of polymers are relatively lower, but can be effectively improved by doping with different types of fillers. Moreover, structural modification is also an effective way to enhance sensor performance. For instance, organic molecules with reversible redox characteristics have been doped into the material structure to improve the sensitivity and signal amplitude of sensors.^25^ Previous studies have also focused on new fabrication methods to improve the polarity of flexible piezoelectric materials, such as ink-jet printing, magnetron sputtering and roll-to-roll processing.

**Fig. 1:**
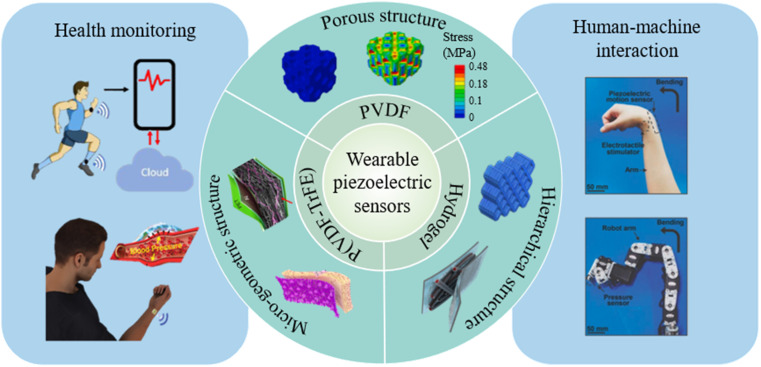
Schematic diagram showing the development of wearable piezoelectric sensors. Sensing materials with porous structures, micro-geometric structures and hierarchical structures. This figure has been reproduced from ref. 27, 28 and 29 with permission from Elsevier, copyright 2020, 2021, 2024; ref. 30 with permission from American Chemical Society, copyright 2021; ref. 31 with permission from Springer Nature, copyright 2022. Applications in health monitoring and human–machine interaction. This figure has been reproduced from ref. 32 and 33 with permission from John Wiley and Sons, copyright 2014, 2023; ref. 34 with permission from Elsevier, copyright 2023.

Recent advances in piezoelectric sensors demonstrate their huge potential and promising prospects in flexible and wearable electronics. Even if previous review articles have focused on the research progress of piezoelectric materials,^22,26^ there is a lack of a comprehensive review on flexible piezoelectric sensors especially concerning material structure engineering and smart wearable applications. In this review, we highlight scientific and technological breakthroughs in wearable piezoelectric sensors, concentrating on polymer materials, microengineered structures, fabrication methods and wearable applications, as shown in Fig. 1. Firstly, basic working mechanisms and design principles of piezoelectric materials and sensors are introduced. Then, a detailed overview of flexible piezoelectric materials is provided, along with the common fabrication techniques. Subsequently, material structure design and engineering strategies are discussed to improve the sensing performance of flexible piezoelectric sensors. After that, smart wearable applications of flexible piezoelectric sensors are provided especially in health monitoring and human–machine interactions. Finally, perspectives on critical challenges and promising principles concerning advanced manufacturing, biological safety and functional integration are offered to provide insights into future research directions of piezoelectric materials and wearable sensors.

## 2 Theory and mechanism of piezoelectric sensors

**Fig. 2:**
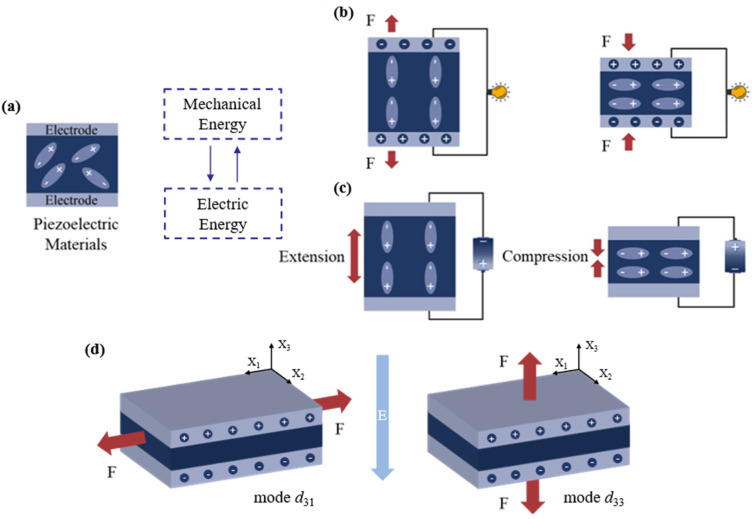
Schematic diagram showing (a) the mechanism of piezoelectric materials; (b) direct piezoelectric effect; (c) converse piezoelectric effect. This figure has been reproduced from ref. 36 with permission from American Chemical Society, copyright 2023. (d) Operating modes of a piezoelectric material: mode *d*_31_ (left) and mode *d*_33_ (right).

Piezoelectricity was first discovered in 1880 by Pierre and Jacques Curie, when they were conducting studies on crystals of quartz, tourmaline and Rochelle salt.^35^ The piezoelectric effect is a physical phenomenon occurring in piezoelectric materials under the action of mechanical force or an electric field, which is caused by the asymmetry of the crystal structure of materials (Fig. 2a). There are two distinct piezoelectric effects. Fig. 2b shows the direct piezoelectric effect, where the applied pressure results in a charge shift inside the piezoelectric crystal, leading to the accumulation of positive and negative charges on the two surfaces in the polarization direction. In the converse piezoelectric effect, which was proven using fundamental thermodynamic principles in 1881,^37^ the application of voltage results in mechanical displacement in the material (Fig. 2c). The constitutive eqn (1) for the direct piezoelectric effect and eqn (2) for the converse piezoelectric effect are given below.

1 *D* = *dT* + *εE*

2 *X* = *sT* + *dE*

where *D* = electrical displacement, *d* = piezoelectric coefficient, *T* = stress, *ε* = permittivity of the material, *E* = electric field, *X* = strain, and *s* = mechanical compliance.

The working principle of piezoelectric sensors is based on the direct piezoelectric effect. Eqn (3) for the charge accumulating at the surface of the piezoelectric material is given below.

3 *Q* = *d*_*mn*_ × *F*_*x*_

where *Q* = charge, *d*_*mn*_ = piezoelectric coefficient, and *F*_*x*_ = the applied force. Piezoelectric materials have two main strain modes, mode *d*_31_ and mode *d*_33_. The first subscript number denotes the poling direction, while the second number is an indication of the direction of the applied force. Piezoelectric performance is related to the angle between the stress and polar axis within materials. As shown in Fig. 2d, mode *d*_31_ involves the application of stress perpendicular to the direction of polarization, while mode *d*_33_ involves the application of stress in the direction of polarization. For the same piezoelectric material, the *d*_33_ value is usually greater than or equal to two times the *d*_31_ value.^21^ Piezoelectricity in organic materials arises from the reorientation of molecular dipoles under mechanical stress, whereas inorganic materials generate piezoelectricity through the displacement of ions within crystals under mechanical stress. Consequently, compared to inorganic materials, organic materials generally demonstrate relatively inferior piezoelectric performance.

## 3 Advances in piezoelectric polymer materials

Piezoelectric polymer materials mainly include PVDF and its copolymers, hydrogels, cellulose, nylon, *etc.* The advantages of polymers such as low density, flexibility, ease of processing and low cost make them ideal choices for wearable electronic devices. Piezoelectricity in polymer materials mainly arises from the reorientation of molecular dipoles, resulting in poorer piezoelectric properties compared to piezoelectric ceramics. However, their low permittivity and low acoustic impedance make them irreplaceable in the field of flexible sensors.^38^ Among them, PVDF and its copolymers are a class of polymers with the best piezoelectric properties, so they are favored by many researchers.^39^ In addition, hydrogels have also emerged as hot piezoelectric materials in recent years, due to their high transparency, excellent biocompatibility, antifreeze performance and other advantages.^3^ The following section will summarize the research progress of these three piezoelectric polymers, focusing on the principles and methods to improve piezoelectric performance. Tables 1 and 2 show the enhanced piezoelectric performance of sensors based on piezoelectric polymers.

**Table 1:** Summary of piezoelectric materials based on PVDF and P(VDF-TrFE) for flexible wearable sensors

Polymer matrix	Filler	Synthesis method	β phase content	Piezoelectric coefficient	Output performance	Sensitivity
PVDF	ZnO nanoparticles^40^	Electrospinning	81.4%	6.38 pC N^−1^	∼3 V, 254 nA	0.37 mV kPa^−1^
	ZnO nanorods^41^	Solvent casting	76.1%	−1.17 pC N^−1^	1.81 V, 0.57 μA	N/A
	PDA-BaTiO_3_ nanoparticles^42^	Electrospinning	N/A	22.56 pC N^−1^	∼14 V, ∼0.53 μA	3.95 V N^−1^
	FD-BaTiO_3_ nanoparticles^43^	3D printing	84.25%	69.1 pC N^−1^	∼30 V, ∼1 μA	61.6 mV kPa^−1^
	TOS-BaTiO_3_ nanoparticles^44^	Screen-printing	86.38%	33.5 pC N^−1^	20 V	45.4 mV kPa^−1^
	GR^45^	Solvent casting	N/A	N/A	0.4 V	3.95 V N^−1^
	MWCNTs^46^	FDM	76.8%	N/A	5.7 V	2.65 V kPa^−1^
	CNTs^47^	Gap spinning	>90%	N/A	∼6.8 V	1.28 V N^−1^
	MWCNTs/GR/MnO_2_ (ref. 48)	Solvent casting	>90%	17–33 pC N^−1^	N/A	N/A
	Zn@C nanoparticles^49^	Electrospinning	88.3%	39.5 pC N^−1^	37 V	0.98 V kPa^−1^
P(VDF-TrFE)	BaTiO_3_ nanoparticles^50^	Electrospinning	81.8%	N/A	17.6 V	N/A
	BaTiO_3_ nanoparticles^51^	3D printing	N/A	20 pC N^−1^	6 V, 2 μA cm^−2^	N/A
	Ag nanowires/Sm-PMN-PT^52^	Solvent casting	N/A	∼32 pC N^−1^	83.5 V	N/A
	ZnSnO_3_-CNT^53^	Electrospinning	N/A	N/A	97.5 V, 1.16 μA	41.7 mV μm^−1^
	CsPbBr_3_-CNT^54^	Electrospinning	92.9%	58.8 pm V^−1^	∼15.9 V, ∼1128 nA	3.31 nA N^−1^
	KNN^55^	Spin coating	56.3%	72.62 pm V^−1^	∼5 V, ∼1 μA	N/A
	GR/KNN^56^	Solvent casting	47.96%	−28.4 pC N^−1^	7.4 V	N/A
	Cu wires^57^	Electrospinning	∼88%	N/A	0.8 V	60.82 mV N^−1^

**Table 2:** Summary of piezoelectric hydrogel materials for flexible wearable sensors

Hydrogel	Filler	Output performance	Sensitivity	Operation range	Response time
Gel/OCS	Surface-aminated BaTiO_3_ (ref. 58)	85.90 mV	49.61 mV kPa^−1^	<1.27 kPa	24 ms
p(NVCL-*co*-DEGDVE)	ZnO^59^	N/A	36 pC N^−1^, 0.14 nC °C^−1^	30–50 °C at 96% RH	28 ms
PAAN	Gly/Zn^2+^/PVDF^60^	60 mV	1.34 mV kPa^−1^	<3 kPa	31 ms
PAN	PVDF^61^	30 mV, 2.8 μA	N/A	N/A	31 ms
Gelatin	PVDF/Ppy^62^	N/A	GF = 27.8	0.1–55 kPa	0.1 s
CHACC	PEDOT:PSS/P (VDF-TrFE)^63^	100 mV	GF = 19.3	5–25 Hz	63.2 ms
Bacterial cellulose	ImClO_4_ (ref. 64)	N/A	4.24 mV kPa^−1^	0.2–31.25 kPa	N/A

### 3.1 Polyvinylidene fluoride (PVDF)-based materials

**Fig. 3:**
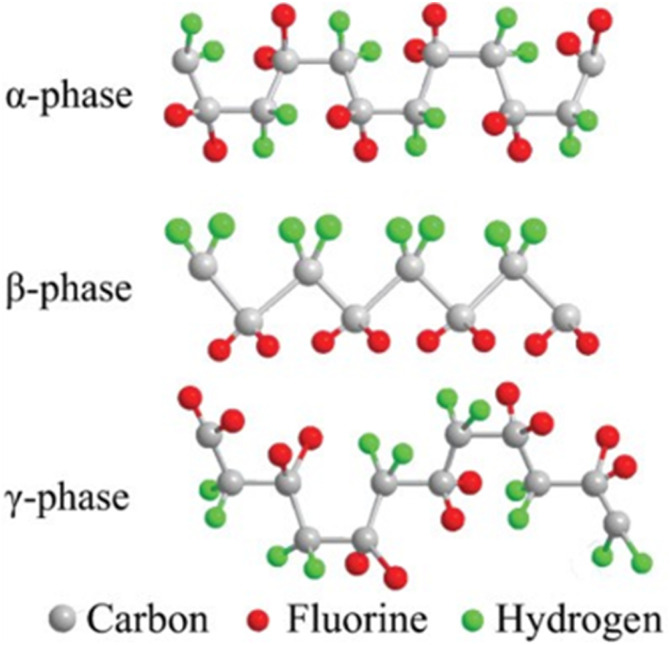
Schematic diagram showing the chain conformations of α, β and γ phases in PVDF. This figure has been reproduced from ref. 66 with permission from John Wiley and Sons, copyright 2018.

The piezoelectric properties of PVDF, a semi-crystalline polymer synthesized by the polymerization of vinylidene fluoride (VDF),^65^ are determined by the orientational polarization of its crystals and the proportion of its polar phase. PVDF has five different phases: α, β, γ, δ and ε. Among them, the α, β, and γ phases are most commonly observed (Fig. 3).^67,68^ The α phase a *trans*-gauche (TGTG′) conformation, which is nonpolar because the dipole moments cancel out each other.^69,70^ The β phase has a planar zigzag conformation of all trans (TTT),^71^ allowing for a large spontaneous polarization as the dipoles align parallel to each other and perpendicular to the molecular chain axis. In all phases, the β phase exhibits the highest dipole moment (7.0 × 10^−30^ cm),^72^ polarizability (131 mC m^−2^)^73^ and electrical activity. The γ phase with a *trans*-gauche (T3GT3G′) conformation is a transitional state between α and β, showing a lower dipole moment (4.0 × 10^−30^ cm) than the β phase. Therefore, to enhance the piezoelectric performance of PVDF, it is critical to maximize the β phase proportion and the polarity. An exhaustive review has been written on obtaining the β phase PVDF.^74–78^ Using different types of fillers to synthesize nanocomposites can change the crystal structure and effectively improve the piezoelectric coefficient of PVDF. Moreover, innovating and improving fabrication methods can also enhance the piezoelectric performance of PVDF. Synthesizing copolymers is another effective method, which will be discussed in detail later.

The inclusion of different kinds of fillers significantly affects the overall properties of the resulting nanocomposites. The formation mechanism of the β phase in PVDF-based nanocomposites can be attributed to either the effective dispersion of nanoparticles or the specific interaction between nanoparticles and the PVDF chain. A number of parameters like polymer chain mobility, chain confirmation, crystallinity, *etc.* have been reported to control the interaction of the nanomaterials with PVDF.^65^ A large number of conducting, non-conducting and hybrid fillers have been used to prepare piezoelectric polymer nanocomposites, such as ZnO,^40,41^ BaTiO_3_,^42,43,79^ graphene (GR),^45,80^ multiwalled carbon nanotubes (MWCNTs),^46^ carbon nanotubes (CNTs),^47,77^ MWCNTs/GR/MnO_2_ hybrids^48^ and carbon-coated zinc oxide nanoparticles.^49^

**Fig. 4:**
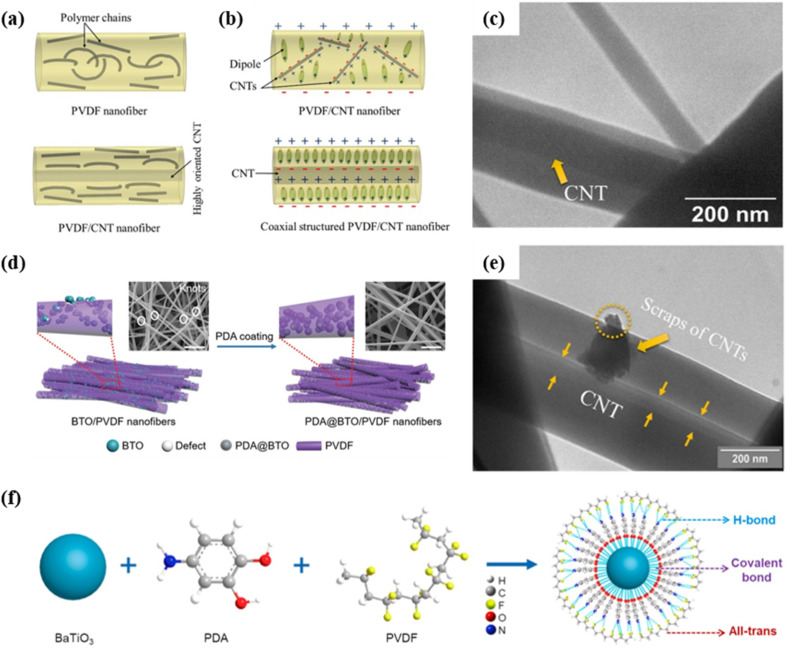
Schematic diagram showing (a) the effect of CNTs on the orientation of polymer chains; (b) the dipoles and inductive charge distribution within PVDF/CNT nanofibers. (c) and (e) Morphology of PVDF/CNT nanofibers. TEM images showing the perfect longitudinal orientation of the CNTs within the PVDF nanofibers: (c) 1.25 wt% and (e) 3 wt%. This figure has been reproduced from ref. 47 with permission from Springer Nature, copyright 2022. (d) Comparisons between unmodified and modified electrospun fibers *via* dopamine coating. This figure has been reproduced from ref. 42 with permission from John Wiley and Sons, copyright 2021. (f) Schematic diagram showing PDA-enabled interface modification between the BaTiO_3_ filler and PVDF matrix. This figure has been reproduced from ref. 79 with permission from Elsevier, copyright 2021.

In 2022, Sharafkhani *et al.* made a breakthrough by preparing a high-performance sensor with a β-phase content exceeding 90%, using PVDF/CNTs nanofibers.^47^ By adjusting the electrospinning conditions, the CNTs achieved preferential orientation along the longitudinal axis of the PVDF nanofibers, rearranging polymeric chains (Fig. 4a). More dipoles with the same orientation were induced on the surfaces of nanofibers and the total number of inductive charges increased (Fig. 4b), which promoted the formation of the β phase. Using a strong electric field, a high extension rate of the spinning jet during electrospinning and a high rotation speed of the gap collector during gap spinning can increase the drawing force and eliminate any remaining irregularities in the structure of nanofibers (Fig. 4c and e). The highest output voltage obtained was 6.8 V for fully oriented PVDF nanofibers incorporated with 1.25 wt% axially aligned CNTs. The output voltage remained almost constant over a long period of 1800 cycles, demonstrating the excellent stability of the sensor. The proper interactions between the aligned PVDF chains and the well-oriented CNTs were responsible for the complete elimination of the nonpolar α-phase, leading to the rapid increase in the degree of the crystallinity, elastic modulus, electrical conductivity and dielectric constant of the system.

The percolation threshold depends on the shapes of the fillers, as well as the polarity, viscosity and degree of crystallization of the polymer. An increased percolation threshold hinders the uniform distribution of the fillers, making surface modification of nanofillers necessary.^65^ Among different fillers, BaTiO_3_ has been commonly studied for surface modification. In 2021, Su *et al.* proposed a muscle-fiber-inspired (MFP) nonwoven piezoelectric textile based on PVDF doped with BaTiO_3_.^42^ Dispersing polydopamine (PDA) into the BaTiO_3_/PVDF nanofibers significantly smoothed the fiber surface and promoted interfacial adhesion and linkage, leading to a stronger electromechanical coupling effect and thus a higher piezoelectric response (Fig. 4d). The MFP sensor exhibited outstanding sensitivity (3.95 V N^−1^) and long-term stability (<3% decline after 7400 cycles), demonstrating its potential for pulse wave measurement and human motion monitoring. Later, he comprehensively investigated the functionality and mechanism of PDA-assisted surface modification.^79^ The cross-linking of PDA around BaTiO_3_ fillers not only improved the modulus match and stress transfer efficiency between the ceramic fillers and polymer matrix, but also facilitated the all-trans conformation in the PVDF chains (Fig. 4f). In 2021, Li *et al.* employed a 3D printing technique to fabricate self-powered wearable sensors based on the hydrophobic surface-functionalized BaTiO_3_/PVDF composite films.^43^ To strengthen the interface bond between BaTiO_3_ and PVDF, the BaTiO_3_ nanoparticles were surface functionalized using hydrophobic 1*H*,1*H*,2*H*,2*H*-perfluorodecyltriethoxysilane. The printed self-powered sensor exhibited excellent sensitivity with a of *d*_33_ valueof 69.1 pC N^−1^, two-fold higher than that of the unfunctionalized BaTiO_3_/PVDF counterpart. Similarly, in another study of Li *et al.*, a flexible and high-performance nanocomposite sensor based on a BaTiO_3_/PVDF composite film *via* screen printing was developed.^44^ Triethoxy(octyl)silane-coated BaTiO_3_ nanoparticles were anchored onto PVDF, in order to minimize the effect of nanofiller agglomeration and assist in the uniform dispersion of fillers. Consequently, the screen-printed device exhibited a significantly enhanced output voltage of 20 V and a higher power density of 15.6 μW cm^−2^, even after 7500 cycles.

Various fabrication methods have been developed to enhance the β-phase of PVDF, including electrospinning, melt spinning, 3D printing, phase transition, quenching and annealing. Among these techniques, electric field poling and stretching are the most typical methods used for the transformation from the α-phase to the β-phase.^81,82^ Poling involves reorienting dipole moments, while stretching aligns PVDF dipole chains with the applied stress direction. Quenching and annealing directly impact the alteration of the crystalline structure, and facilitate low-temperature nucleation at high quenching rates to induce the formation of the β-phase.^83,84^ Although other commonly used fabrication methods such as solvent casting and spin coating are simple to operate, they mainly result in α-phase formation of PVDF, necessitating subsequent poling treatment. Considering the increasing popularity of electrospinning, melt spinning and 3D printing in recent years, a comprehensive discussion on them will be provided below.

**Fig. 5:**
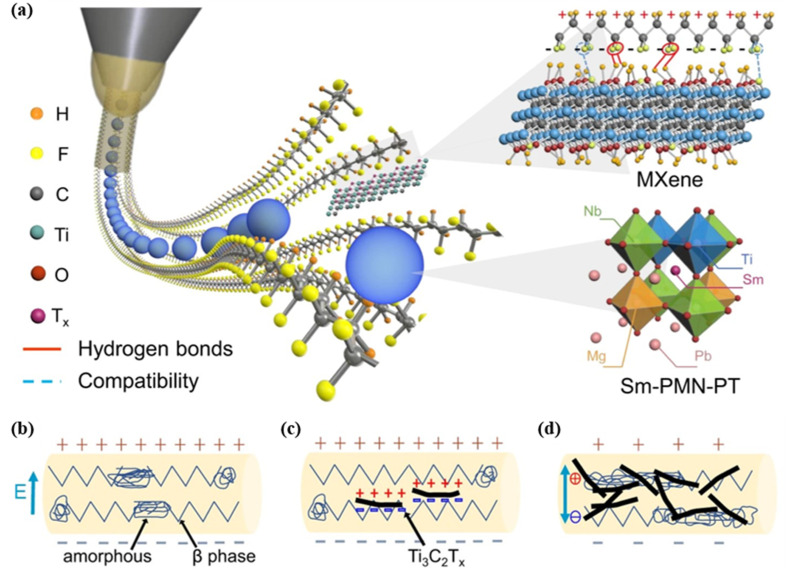
(a) Schematic diagram showing the electrospinning procedure for piezoelectric nanofiber synthesis. Inset: intermolecular interaction between MXene and PVDF; and a 2 × 2 × 2 supercell of Sm-PMN-PT as a ceramic filler. A schematic diagram showing the polarization process of electrospun nanofibers doped with (b) no MXene, (c) appropriate amount of MXene and (d) excessive amount of MXene. This figure has been reproduced from ref. 88 with permission from Springer Nature, copyright 2022.

Electrospinning is a promising fabrication method using electric force to create fine fibers from a polymer solution. In the process, because the Coulomb and gravitational forces are greater than the surface tension, spinning polymer droplets gradually form a thin jet that solidifies into a fiber in the receiving plate. Electrospinning produces PVDF nanofibers with a high β phase fraction and crystallinity by aligning molecular dipoles along the applied voltage direction. Various properties of nanofiber films can be well-controlled by adjusting spinning parameters like voltage, needle diameter, solvent, viscosity, temperature and relative humidity. Compared with other electrospinning methods, solution electrospinning has the irreplaceable advantage of directly forming β-phase PVDF .^78,85^ In recent years, the needleless techniques have emerged rapidly for effectively increasing the productivity rate of nanofibers.^86,87^ Su *et al.* fabricated a high-performance piezoelectric composite *via* termination engineering using Ti_3_C_2_T_*x*_ MXene templating, which could lead to an output gain of 160%.^88^ The hydrogen bond anchoring strategy, wherein MXene manipulates the intermolecular interactions within the all-trans conformation of a polymer matrix was translated into reality successfully *via* electrospinning (Fig. 5a–d). In 2023, Feng *et al.* explored the effects of nanoparticles (ZnO, BaTiO_3_ and SrTiO_3_) and core–sheath structures on the PVDF electrospun fibers.^89^ The results showed that nanoparticles could be used as a nucleating agent to increase the content of the β phase in fibers, while destroying the continuity of the fibers. And the core–sheath structure could significantly improve the mechanical property loss caused by nanoparticles and had no effect on the β phase and piezoelectric constants.

**Fig. 6:**
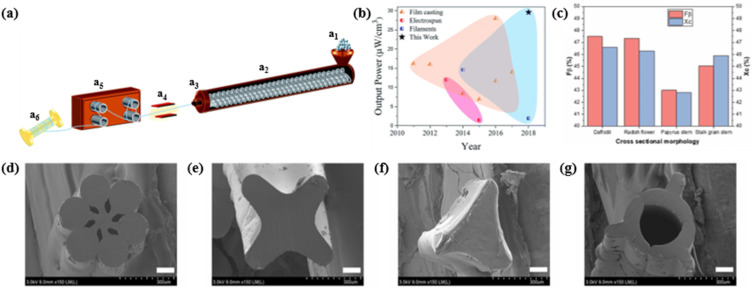
(a) Schematic diagram showing the melt-spinning set-up to produce continuous PVDF fibers: (a_1_) PVDF powder feed; (a_2_) twin screw extruder; (a_3_) single hole spinneret; (a_4_) heating zone for slow cooling as-spun fibers; (a_5_) stretching zone and (a_6_) take up, resulting in a spool of melt-spun PVDF piezoelectric fibers. (b) Comparison of the power density for a triaxial braided energy harvesting generator and previously reported energy generators based on pure piezoelectric PVDF films and fibers. This figure has been reproduced from ref. 91 with permission from Royal Society of Chemistry, copyright 2019. (c) Calculated β-phase fractions and degree of crystallinity for the PVDF fibers based on different cross-sectional morphologies. SEM images showing PVDF fibers through melt spinning associated with biomimetic cross-sectional morphologies: (d) daffodil, (e) radish flower, (f) papyrus stem and (g) stalk grain stem. This figure has been reproduced from ref. 92 with permission from Elsevier, copyright 2022.

Melt spinning is another widely employed manufacturing technique for piezoelectric fibers, enabling efficient large-scale fabrication of filaments with consistent morphology, thickness and strength. This is difficult to achieve with electrospinning.^90^ More importantly, melt spinning is more environmentally friendly as it does not require organic solvents. The polymer powder is melted and pumped through a spinneret with holes. The extruded filaments undergo post-processing through various techniques, such as cold drawing and poling, to optimize their piezoelectric performance before being collected on a bobbin (Fig. 6a). Notably, a recently proposed rheological prediction method, based on the Rouse relaxation time, could directly generate piezoelectric fibers with flow-induced high crystal orientation from the melt, potentially optimizing melt spinning into a one-step process.^93,94^ The manufactured piezoelectric filaments exhibit sufficient flexibility for integration with weaving techniques, enabling the formation of diverse structures such as braiding, knitting weaving and coiling.^95^ Mokhtari *et al.* proposed a new strategy combining piezoelectric fibers, conductive fibers and braiding technology.^91^ The developed triaxial textile energy harvester used high-performance PVDF fibers as the piezoelectric polymer and silver coated nylon as inner and outer electrodes. The triaxial braided structure allowed poling between the inner and outer electrodes with PVDF fibers as the intermediate structure, promoting radial poling along the fiber. This significantly enhanced power output compared to previously reported energy generators based on pure PVDF films and fibers (Fig. 6b). This strategy also addressed the stability issue caused by poor fatigue resistance of metal electrodes, showing extreme durability. In 2022, Kim *et al.* prepared melt-spun PVDF fibers with plant-inspired cross-sectional morphologies by easily changing the spinneret,^92^ as depicted in Fig. 6d–g. The daffodil flower-shaped PVDF fiber exhibited the best piezoelectric performance, attributed to its high surface area increasing the β-phase fraction and its high contact volume maximizing the active area for piezoelectricity generation (Fig. 6c). The piezoelectric performance was also influenced by the morphology of BaTiO_3_ fillers. In comparison to nanoparticles, the rod morphology more effectively induced asymmetry in the fiber during the winding process and polarization during the poling process.

Compared to traditional fabrication approaches, 3D printing, featuring a layer-by-layer processing procedure, significantly reduces the cost of handling complex geometric shapes and obtaining elaborate structures. One method regarding 3D printing is solvent evaporation assisted (SEA) 3D printing,^96,97^ a low-temperature and low-energy deposition method. Another method of 3D printing is fused deposition modeling (FDM),^98^ which improves the diffusion of nanoparticles in polymer matrix nanocomposites and prevents nanoparticle agglomeration. In the study by Li *et al.*, hydrophobic surface-functionalized BaTiO_3_/PVDF composite films for a self-powered sensor were prepared by 3D printing.^43^ Due to the special mortise-tenon joint structure in the nanocomposite film, the printed piezoelectric sensor exhibited high sensitivity (61.6 mV kPa^−1^), excellent mechanical durability and stability after 20 000 cycles. In 2021, Song and his colleagues developed a PVDF energy harvester with complex 3D bioinspired bone structures, *via* an *in situ* chemical foaming assisted FDM 3D printing method.^98^ The porous biomimetic bone structure magnified the stress–strain effect, improved the output capacity (a voltage of 13 V and a maximum current density of 0.27 μA cm^−2^) and enhanced the piezoelectric performance (86.72% β-phase content and a *d*_33_ of 69.1 pC N^−1^ ).

### 3.2 Polyvinylidene fluoride-trifluoro ethylene (P(VDF-TrFE))-based materials

**Fig. 7:**
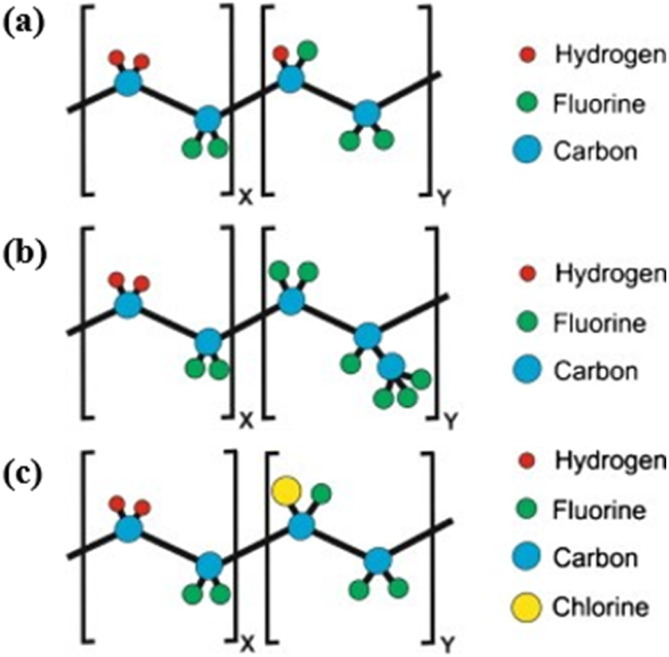
Schematic diagram showing the repeat units of (a) P(VDF-TrFE); (b) P(VDF-HFP); and (c) P(VDF-CTFE). This figure has been reproduced from ref. 75 with permission from Elsevier, copyright 2014.

Compared to PVDF-based materials, copolymers synthesized by incorporating PVDF consistently exhibit superior piezoelectric performance. Over the past few decades, a diverse range of comonomers have been utilized, including trifluoroethylene (TrFE), hexafluoropropylene (HFP)^99–101^ and chlorotrifluoroethylene (CTFE)^102–104^ (Fig. 7a–c). The introduction of these monomers modifies the molecular structure, inducing a conformational transition from *trans*-gauche to all-trans. The transition reduces the activation energy necessary for the transformation from the nonpolar phase to the polar phase, making it easier for copolymers to crystallize into the β-phase at ambient temperature. However, low crystallinity affects electroactivity and limits piezoelectricity. The performance of PVDF copolymers can be optimized by adjusting the monomer content, as well as through polarization methods such as stretching, annealing and electric field.^105–107^ Among these copolymers, P(VDF-TrFE) copolymers with high piezoelectric coefficients (*d*_33_ = −30 to −40 pC N^−1^ and *d*_31_ = 25 pC N^−1^)^21^ have garnered substantial research attention, which will be comprehensively discussed subsequently. Compared to other copolymers, P(VDF-HFP) copolymers exhibit a higher *d*_31_ attributed to the reversible transformation between a poled α-like structure and β-like structure.^108^ P(VDF-CTFE) could demonstrate a remarkable *d*_33_ reaching up to 140 pC N^−1^ through electric field polarization.^109^

**Fig. 8:**
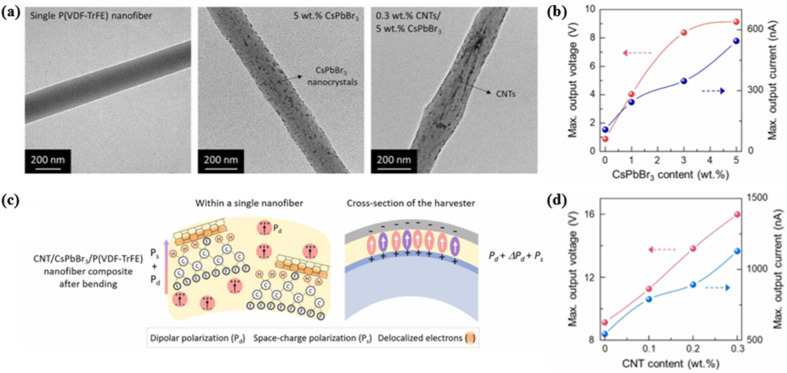
(a) TEM images of single P(VDF-TrFE) nanofibers (left) without inclusions, (middle) with 5 wt% CsPbBr_3_ nanocrystals and no CNTs, and (right) with 0.3 wt% CNTs and 5 wt% CsPbBr_3_. (c) Schematic diagram showing the contributions by CsPbBr_3_ and CNTs to the electric polarization of P(VDF-TrFE) nanofiber composite structures. The peak value line plot of output voltage and output current for the composites with different contents of (b) CsPbBr_3_ nanocrystals and (d) CNTs. This figure has been reproduced from ref. 54 with permission from Elsevier, copyright 2022.

One of the widely used methods for improving the piezoelectric properties is to dope different kinds of functional fillers into polymers, which increases the β-phase content in the crystal region of P(VDF-TrFE). A variety of materials have been successfully incorporated with P(VDF-TrFE) at different concentrations and configurations, such as BaTiO_3_,^50,51,110^ silver nanoparticles,^52,111,112^ CNTs^53,54^ and potassium sodium niobate (KNN).^55,56^ It is worth noting that these fillers may induce a strong decay in crystallinity of P(VDF-TrFE) if they are not well dispersed and oriented in the matrix.^113^ In 2022, Han *et al.* selected CNTs and inorganic perovskite CsPbBr_3_ nanocrystals as fillers for a piezoelectric P(VDF-TrFE)-nanofiber harvester.^54^ The electrospinning technique was used to fabricate high-quality nanofiber composites, wherein the fillers were well dispersed within the nanoscale fibers (Fig. 8a). The combined effects of halide nanocrystals and CNTs more effectively improved the harvesting performance, achieving a maximum voltage of 15.9 V and a current of 1128 nA (Fig. 8b and d). The apparent enhancement was associated with the creation of additional dipolar polarization Δ*P*_d_ resulting from the β phase and halide, and interfacial polarization P_s_ induced by CNTs. Fig. 8c illustrates the mechanism of induced polarization under the bending motion. The optimal pressure sensor was further applied in signal-mapping and intelligent health-monitoring systems to recognize selective discrete gait motions and finger or foot pressing. Kang *et al.* proposed a peapod-inspired design in which ZnSnO_3_ anchored on surface-modified CNTs (SMCs) allowed significant enhancement of piezoelectricity of the P(VDF-TrFE)-based nanofibers.^53^ As a non-centrosymmetric oxide, the large displacement of Zn along the *z*-direction in the ZnO_6_ cluster of ZnSnO_3_ facilitated spontaneous polarization and a strong piezoelectric response. The unique structure of the ZnSnO_3_-decorated SMCs promoted electron transfer from ZnSnO_3_ nanoparticles to the electrically conductive SMCs. Pulsed laser ablation was an effective method for modifying the CNT surfaces, ensuring that the interface between ZnSnO_3_ and SMC did not show any structural defects or pores. The developed piezoelectric device exhibited exceptional piezoelectric sensitivity, capable of detecting imperceptible pulses even in posterior tibial arteries.

**Fig. 9:**
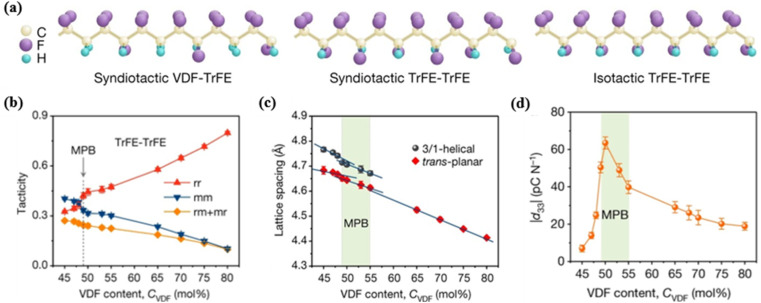
(a) Schematic diagram showing the chain tacticity in P(VDF-TrFE). (b) Tacticity of the TrFE–TrFE segment as a function of VDF content. Data were given for the isotactic (mm), syndiotactic (rr) and heterotactic (mr + rm) configurations. (c) Intermolecular lattice spacing *versus* VDF content. The light-green-shaded area indicated the transition region, across which the structure changed abruptly. (d) Magnitude of *d*_33_ as a function of VDF content. This figure has been reproduced from ref. 114 with permission from Springer Nature, copyright 2018.

Very recently, a noteworthy approach using the behavior reminiscent of morphotropic phase boundary (MPB) characteristics of P(VDF-TrFE) has been proposed. MPB refers to the vertical phase boundary of a certain phase diagram where two or more phases can especially coexist, which is widely utilized in piezoelectric ceramics. In 2018, Liu *et al.* reported the stereochemically induced behavior in ferroelectric P(VDF-TrFE) copolymers, similar to the MPB in perovskites,^114^ introducing this concept into piezoelectric polymers for the first time. The increase in the molar fraction of the chiral monomer TrFE caused the intramolecular order-to-disorder transition because of the lack of chain tacticity (Fig. 9a and b). The conformational competition between the *trans*-planar phase and the 3/1-helical phase drove the formation of MPB-like transition region (Fig. 9c), where normal ferroelectricity coexisted with relaxor ferroelectricity with 49 mol% ≤ VDF ≤ 55 mol%. At VDF = 50 mol%, the piezoelectric coefficient reached an anomalous maximum of −63.5 pC N^−1^ (Fig. 9d), which is twice the previous results.^115^ The significantly enhanced piezoelectric properties were attributed to the polarization rotation between energetically degenerate *trans*-planar and 3/1-helical phases.^116^ Park *et al.* developed an energy harvesting device fabricated from MPB-associated P(VDF-TrFE) copolymer nanofibers through electrospinning.^117^ The unpoled device with TrFE = 50 mol% showed superior output performance compared to that of the poled device with TrFE = 30 mol%, indicating the overwhelming energy harvesting ability of MPB-like P(VDF-TrFE) nanofibers. In 2022, Han *et al.* successfully enhanced the piezoelectricity in a series of solution-cast P(VDF-TrFE-CTFE) terpolymer thin films *via* creating mixed ferroelectric and relaxor phases.^118^ An increasing concentration of CTFE was observed to promote the formation of a 3/1-helical phase, and a behavior reminiscent of the MPB emerged when the CTFE content ranged from 1.7 to 5.0 mol%. Specifically, the terpolymer consisting of VDF/TrFE/CTFE in a molar ratio of 64.5/33.1/2.4 exhibited a *d*_33_ value of −55.4 pC N^−1^, representing an 85% increase compared to the well-known P(VDF-TrFE) copolymer with a molar ratio of 65/35.

**Fig. 10:**
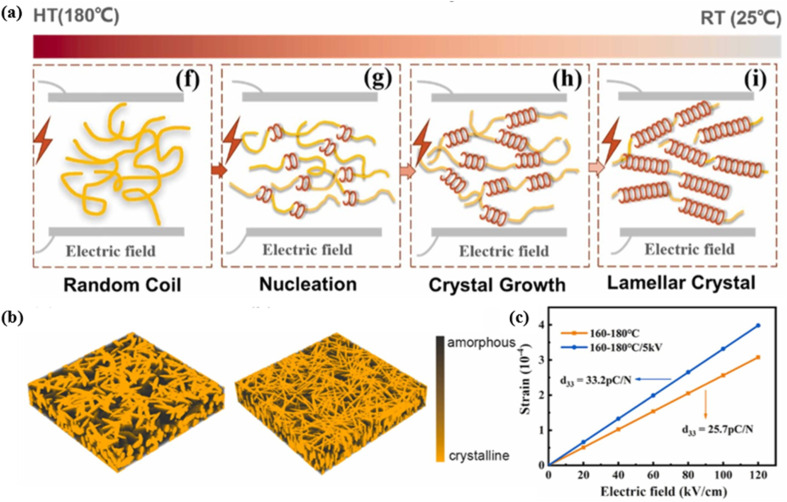
(a) Schematic diagram showing the proposed mechanism for *in situ* electrostatic field regulating the recrystallization behaviors of P(VDF-TrFE) films. (b) Simulation microstructure models of (left) 160–180 °C and (right) 160–180 °C/5 kV P(VDF-TrFE) films. (c) The simulated average strain–electric field line plot for the two microstructures. This figure has been reproduced from ref. 125 with permission from Elsevier, copyright 2022.

Commonly used fabrication methods to improve the piezoelectric performance of P(VDF-TrFE) polymers include electrospinning,^57,119^ printing,^51,120,121^ epitaxy growth^122^ and solvent choice.^123,124^ Electrospinning can not only increase the crystallinity of electroactive β phases, but also provide a promising strategy for morphological design. Lu *et al.* developed piezoelectric microfibers with a novel core–sheath structure by directly electrospinning P(VDF-TrFE) onto a flexible conductive wire.^57^ Precise control of fiber diameter and thickness of the P(VDF-TrFE) functional layer was achieved. The smart fiber exhibited a high sensitivity of 60.82 mV N^−1^ and excellent durability over more than 15 000 cycles under positive compression. The fabricated fiber also showed extreme flexibility, being able to bear severe deformations such as bending and knotting. In 2022, Lv *et al.* prepared the P(VDF-TrFE) films with enhanced piezoelectric performance by applying an *in situ* electrostatic field during the thermal annealing process.^125^ The electrostatic field could regulate the recrystallization behaviors of P(VDF-TrFE) films, promoting the generation of nucleation sites for the polar β lamellae crystals, resulting in a high degree of crystallinity reaching 62.9% and almost 100% polar β-phase. Recrystallization occurred as the temperature dropped just below the crystallization point (150.4 °C), with increased crystallinity and decreased particle size *via* the *in situ* electrostatic field (Fig. 10a). As shown in Fig. 10b, two simulation microstructure models were constructed based on AFM images of P(VDF-TrFE) films prepared at 160–180 °C and 160–180 °C/5 kV. The simulated effective *d*_33_ was 25.7 pC N^−1^ for the left structure with large crystal size and low crystallinity, and 33.2 pC N^−1^ for the right structure with small crystal size and high crystallinity, which agreed well with the experimental measurements (Fig. 10c). The piezoelectric sensors exhibited high output power (16 V of voltage and 1.50 nA of current at 50 kPa) and excellent sensitivity (∼167.12 mV kPa^−1^) in the pressure range of 25 kPa to 75 kPa.

### 3.3 Conductive hydrogel-based materials

Hydrogels are polymeric materials with a three-dimensional elastic crosslinked network structure. They are able to hold large amounts of water, resulting in distinct physical and chemical properties. In recent years, conductive hydrogels have received great attention in flexible wearable sensors owing to their excellent flexibility, stretchability and biocompatibility. Conductive hydrogels can be prepared by either bonding the hydrogel network with conductive fillers inherently, such as conductive polymers,^63,126^ carbon-based materials^127,128^ and metal nanoparticles,^58,129^ or compositing the hydrophilic matrix with ionic pendant groups or salts.^130^ Moreover, designing and optimizing the three-dimensional network or composition of polymers can easily imbue conductive hydrogels with additional properties, including self-healing, self-adhesive and anti-freezing capabilities.^60,64,131,132^ Most of the current hydrogel-based sensors are piezoresistive, showing disadvantages including significant hysteresis effects, low mechanical durability, susceptibility to temperature changes and the prerequisite of an external power supply. These disadvantages limit their broad applications. Sensors based on the piezoelectric sensing principle, as a promising alternative solution, have extremely low power consumption and can independently provide electric signal outputs. There are two main types of piezoelectric hydrogel sensors: one is based on the piezoelectric effect and the other is based on the piezoionic effect.

**Fig. 11:**
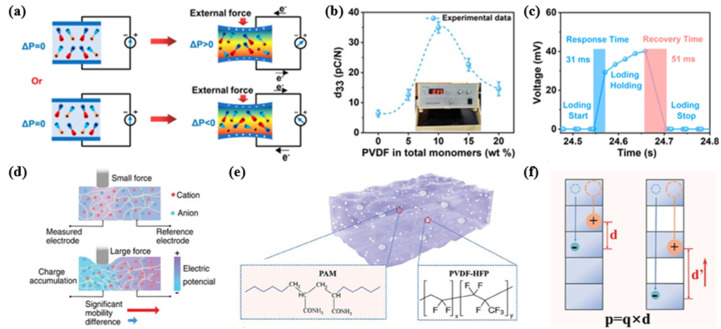
(a) Schematic diagram showing stress-induced polarization generating electrical signals in an external circuit. (b) Piezoelectric constant *d*_33_ of the PAAN/Gly/Zn^2+^ organohydrogels. (c) Response and recovery time of the organohydrogel. This figure has been reproduced from ref. 60 with permission from American Chemical Society, copyright 2024. (d) Schematic diagram showing the mechanism of the piezoionic effect induced by force. This figure has been reproduced from ref. 133 with permission from John Wiley and Sons, copyright 2024. (e) Schematic diagram showing the PVDF-HFP/PAM composite hydrogel. (f) The illustrated contribution of hydrogel porosity to the enhanced electric field generation capabilities. This figure has been reproduced from ref. 134 with permission from John Wiley and Sons, copyright 2024.

Producing a composite piezoelectric hydrogel *via* the integration of electroactive ceramic materials or polymer fillers, such as ZnO,^59,129^ BaTiO_3_,^58^ PVDF^60–62^ and P(VDF-TrFE),^63^ is a prevalent method. Fu *et al.* reported a simple yet highly effective strategy to prepare a self-powered hydrogel with a skin-like Young's modulus (1.33–4.24 MPa), stretchability (175%) and high toughness (1.23 MJ m^−2^).^61^ Incorporating a tough polyacrylonitrile (PAN) hydrogel with PVDF, a maximum *d*_33_ of 30 pC N^−1^ was achieved for the self-powered hydrogels. The dipolar interactions between the PVDF and PAN chains were responsible for the enhanced piezoelectric performance, causing an increase in the β phase percentage of PVDF from 0 to 91.3%. This tough gel was capable of generating an electrical signal output (30 mV voltage and 2.8 μA current) with a rapid response (31 ms). On this basis, Fu and his colleagues conducted further research and synthesized a kind of piezoelectric organohydrogel that integrated piezoelectricity, low-temperature tolerance, mechanical robustness and stable electrical performance.^60^ Using PVDF, acrylonitrile (AN), acrylamide (AAm), *p*-styrenesulfonate (NaSS), glycerol and zinc chloride, the PAAN/Gly/Zn^2+^ organohydrogel was successfully developed *via* a one-pot method and solvent displacement. The organohydrogel with a high *d*_33_ of 35 pC N^−1^ exhibited repeatable and consistent electrical signals for external mechanical stimulation, showing a high voltage output (40 mV), fast response time (31 ms), short recovery time (51 ms) and high sensitivity (1.34 mV kPa^−1^) (Fig. 11b and c). The self-powering mechanism of the organohydrogel can be explained by the stress-induced polarization effect. When stimulated externally, the dipoles inside the organic hydrogel rotated from their equilibrium position, leading to a zero net dipole moment (Δ*P* > 0 or Δ*P* < 0), resulting in a piezoelectric potential difference between the two ends of the organohydrogel (Fig. 11a). The strong hydrogen bonding between glycerol and water molecules suppressed ice formation and therefore endowed the organohydrogel with exceptional anti-freezing ability at a temperature as low as −20 °C. In addition, the organohydrogel exhibited high elongation (780%) and high toughness (8.23 MJ m^−3^) achieved through the synergy of the dipole–dipole interactions and amide hydrogen bonds. The organohydrogel demonstrated sensitive sensing capabilities for finger bending, elbow bending, speaking and pulse beating, as well as gesture signal acquisition.

When an external force is applied, cations and anions migrate within the hydrogel, establishing an ion gradient that leads to charge redistribution and voltage generation (Fig. 11d). This phenomenon constitutes the fundamental principle underlying the piezoionic effect. The key to optimizing piezoionic sensors lies in amplifying the disparity in migration rates between cations and anions; a greater difference in migration rates results in a stronger piezoelectric response. This is determined by factors including ion size, charge, polarity and concentration.^135,136^ Moreover, the composition and morphology of hydrogel networks also play a crucial role. By incorporating nanoparticles and other polymers into hydrogels, as well as adjusting the concentration of electrolyte salts or crosslinking agents, it is feasible to regulate the interactions between different ions and the polymer matrix.^133^ In 2022, Dobashi *et al.* designed an indentation experiment to explore the molecular origins of the piezoionic effect and investigate its applications in sensing.^137^ The open-circuit voltage or short-circuit current was measured between the deformed and undeformed portions of several kinds of hydrogels. The prepared piezoionic skins could detect the touch of a finger on skin, joint flexion and extension. Lu and his colleagues developed a porous and phase-blending hydrogel structure for effective piezoionic electricity generation, electric voltage reaching 600 mV in response to mechanical force.^134^ Introducing the hydrophobic PVDF-HFP polymer into the porous PAM hydrogel *via* solvent displacement remarkably enhanced piezoionic efficacy, due to appropriate porosity and microscopic hydrophobic-hydrophilic phase blending (Fig. 11e). Porosity not only enhanced the deformability of the hydrogels, thus increasing their sensitivity in force-electricity conversion, but also enlarged the cation–anion separation distance, thus enhancing the dipole moment (Fig. 11f). The ionic species moved preferably inside the hydrophilic domain, and for this reason the transition paths in the phase-blending hydrogel significantly narrowed under mechanical stimuli. This caused more significant charge separation and higher piezoelectric voltages. Preliminary applications in mimetic tactile sensors, neural stimulation, enzyme immobilization, sample pretreatment for fast detection were demonstrated.

Compared to hydrogels based on electronic conductors, ionic hydrogels show greater potential for wearable piezoelectric sensors due to their ionic conductivity, flexibility and organizational similarity. Devices based on the piezoionic effect have been shown to produce a wide temporal range of transient signals and provide a much higher charge density than conventional piezoelectric and triboelectric devices.^137,138^ Piezoionic sensors also perform well during electro-mechanical conversions of low-frequency motion, avoiding significant power losses like piezoelectric sensors.^139,140^ The performance of piezoionic sensors is highly controllable *via* a rational investigation into the selection of ions with different sizes and charges. However, the issue of ion leakage remains a significant challenge for piezoionic hydrogels. This problem can result in unstable or even deteriorated sensing performance and cause environmental pollution. Therefore, it is imperative for future research to optimize encapsulation techniques to ensure stability under diverse environmental conditions and extend the lifespan.

## 4 Advances in structural modification of piezoelectric materials

In addition to synthesizing new composite piezoelectric materials, microstructure design and surface modification of the sensing layer represent effective and widely utilized approaches for enhancing sensor performance. Various performance parameters have been shown to be greatly improved by many research groups, including sensitivity, stretchability, response time, the limit of detection and durability. Although several reviews have summarized progress in morphological engineering of flexible pressure sensors, most only focus on piezoresistive and capacitive sensors,^141,142^ or emphasize stretchable structures^143^ without providing a comprehensive overview of structural modifications for sensors with enhanced piezoelectric performance. Therefore, the following section will discuss piezoelectric sensors focused on the commonly used microstructures in detail, such as porous structures, micro-geometric structures and hierarchical structures. Table 3 shows the performance parameters of flexible piezoelectric sensors with different microstructures.

**Table 3:** Summary of flexible piezoelectric sensors with microstructures

Piezoelectric material	Microstructure	Synthesis method	Sensitivity	Operation range	Durability (cycle)	Response/recovery time
PVDF/MXene^27^	Porous	Vapor-induced phase separation	11.9 nA kPa^−1^, 1.4 nA kPa^−1^	<2.5 kPa, 2.5–100 kPa	5000	53/73 ms
P(VDF-TrFE)^119^	Porous	Nonsolvent-induced phase separation, electrospinning	N/A	N/A	5000	N/A
Paper/PVDF/BaTiO_3_ (ref. 144)	Porous	Blade coating	0.13 V kPa^−1^	0–20 kPa	1000	78 ms
P(VDF-TrFE)/PBDMS^145^	Porous	Solvent casting	N/A	1–7 Hz	6600	54 ms
CNC/PEG/GR^146^	Porous	Directional freezing	1.16 V kPa^−1^	N/A	1400	N/A
PDMS/Au-PP^31^	Micro-pyramids	Electrospinning self-assembly	19 kPa^−1^ (≤200 Pa)	0.05 Pa-2.3 kPa	1000	<0.8 ms
P(VDF-TrFE)^147^	Micro-pyramids	Solvent casting, template method	1.62 V kPa^−1^ (100 Pa-9 kPa)	15 Pa-9 kPa, 0–700 Hz	N/A	15 ms
PVDF/PEO^30^	Micro-ribbon	Melt extrusion and leaching	0.092 V N^−1^, 0.034 V N^−1^	<10 N, 10–50 N	6000	70 ms
PDMS/ZnO^148^	Hemispheres	RF sputtering	N/A	N/A	500	N/A
PDMS/PVDF^149^	Biomimetic fish lateral line	3D printing, laser etching	0.24 V N^−1^	>0.0005 N	4000	4 ms
PVDF/ZnO^28^	Hierarchically interlocked	Electrospinning, RF sputtering	3.12 mV kPa^−1^	1.8–451 kPa	5000	55/75 ms
PZT^29^	Hierarchically porous	Freeze casting, DIW	8.98 V kPa^−1^	N/A	N/A	N/A
PVDF/NaCl^150^	Hierarchically porous	Hot compression, salt leaching	5.92 V N^−1^	N/A	N/A	250 ms
PVDF/BaTiO_3_ (ref. 151)	Hierarchically porous	Salt-template method	12.83 V N^−1^	N/A	1600	190 ms
PZT^152^	Microporous, Macro pillar arrays	Freeze casting	1222 V MPa^−1^, 666 V MPa^−1^	∼0.3 MPa, 0.4–0.7 MPa	5000	8.1 ms
ZnO^153^	Asymmetric hollow hemispheres, Macro-porous	Thermal decomposition, Oblique angle deposition	1.64 kPa^−1^	N/A	N/A	N/A
PDMS/BCZT^154^	Microporous, hierarchical droplet-shaped	Freeze casting	1.9 V N^−1^, 8900 mV per strain, 18 mV per degree	N/A	5000	24 ms
PVDF/ZnO^155^	Nanoneedle, hexagonal vertical grown pyramids	Spin coating	N/A	>4 Pa	1000	∼120 ms
P(VDF-TrFE)/BaTiO_3_ (ref. 156)	Sandwich-like	Electrospinning	21.32 mV kPa^−1^	40 kPa	2000	N/A
PDMS/ZnO^157^	Nanowire arrays, hierarchically interlocked	Dip coating, sputter coating	−6.8 kPa^−1^	>0.6 Pa, >57 dB	1000	<5 ms

### 4.1 Porous structures

Porous structures are finding wide use in the current structural design of piezoelectric sensors, especially those based on foam, sponge, aerogel, paper and textiles.^98,144,158,159^ Compared to planar structures, 3D porous structures have a larger specific surface area, increasing the contact area in response to applied pressure to improve sensitivity. The enhanced sensitivity can also be attributed to the introduction of air with low relative permittivity into piezoelectric materials. This results in reducing the permittivity while maintaining a high piezoelectric coefficient. Both stress-induced deformability and stress concentration effects of internal pores endow porous structure sensors with the ability to generate greater piezoelectric output.^146,160^ In addition, porous materials have a high electromechanical coupling factor due to the strong strain response and regular charge distribution after poling.^146,161^ Some sensors with porous structures have also been proven to reduce Young's modulus and effectively improve stress transfer.^98,145^

**Fig. 12:**
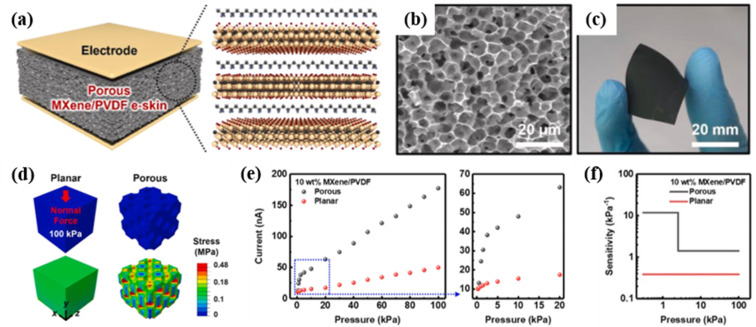
(a) Schematic diagram showing the porous MXene/PVDF e-skin. (b) SEM image of the porous MXene/PVDF e-skin. (c) Photograph of the highly flexible porous MXene/PVDF e-skin. (d) FEA of planar and porous structures and stress distribution under 100 kPa. (e) Comparison of piezoelectric sensing performance between porous- and film-structured 10 wt% MXene/PVDF e-skins. (f) Piezoelectric sensitivity analysis of porous- and film-structured MXene/PVDF e-skins. This figure has been reproduced from ref. 27 with permission from Elsevier, copyright 2021.

In 2021, Kim *et al.* demonstrated self-powered piezoelectric e-skins based on 3D porous structures of MXene (Ti_3_C_2_T_*x*_)/PVDF developed *via* a facile vapor-induced phase separation method (Fig. 12a–c).^27^ The porous structured e-skins permitted enhanced deformation and localized stress concentration on the internal pores, yielding highly sensitive and stable piezoelectric outputs. Fig. 12d shows the finite-element analysis (FEA) of the structural deformation and localized stress distribution in porous structures under 100 kPa compared with the planar structure, clearly explaining the significant increase in piezoelectric sensitivity. As a consequence, the porous e-skins exhibited piezoelectric sensitivities of 11.9 and 1.4 nA kPa^−1^ in low (<2.5 kPa) and high (2.5–100 kPa) pressure ranges respectively, which were 31 and 3.7 times higher than those of planar MXene/PVDF e-skins (0.4 nA kPa^−1^) (Fig. 12e and f). The high-performance piezoelectric e-skins enabled the detection of high-frequency dynamic forces of acoustic sounds and surface textures, as well as low-frequency stimuli of radial artery pulses. In 2023, Yu *et al.* prepared a paper-based piezoelectric sensor with a porous structure and hydrophobic properties by combining flower-like BaTiO_3_ particles with PVDF.^144^ The constructed sensor had a sensitivity of 0.13 V kPa^−1^, a pressure range of 0–20 kPa, a response time of 78 ms and the ability to sense at least 0.1 mg external micro pressure, exhibiting great potential for detecting micro pressure and human motion sensing. The 3.52 V voltage generated by the paper-based sensor was higher than that of the non-paper sensor, which could be attributed to the irregular porous structure of the paper-based device.

Commonly used methods for preparing porous sensing layers include the template method,^150,158,159^ deposition,^98,160^ phase separation^27,119^ and solvent removal.^145,146,162^ These methods provide facilitative tools for researchers to design specific porous structures and effectively tailor their properties. Li *et al.* achieved the orientation pore regulation in porous materials *via* changing the freezing temperature during the freeze-drying of hydrogels to remove ice crystals.^146^ The higher freezing temperature corresponded to smaller porosity and larger pore size. When the length–diameter ratio of porous material pores was increased from 1.1 to 3.3, the voltage output could reach 0.7 V at a moderate ratio. In the study of Kräuter *et al.*, porous ZnO thin films were obtained *via* the calcination of molecular layer-deposited (MLD) ‘zincone’ layers, where the open porosity of films depended on the calcination temperature as well as on the MLD process temperature.^160^ The maximum open porosity of ZnO deposited at 110 °C ranged from 14.5% to 24%, rising with increasing calcination temperature.

### 4.2 Micro-geometric structures

Over the years, researchers have focused on successfully introducing various micro-geometric structures into the piezoelectric materials, including micro-pyramids,^31,147,155,163^ micro-pillars,^164^ micro-ribbons,^30^ and wavy,^165^ hemispherical^148,153^ and biomimetic structures.^98,149,154^ Among them, micro-pyramid structures are the most commonly used. Compared to the cylindrical and hemispherical microstructures, micro-pyramid structures induce greater local stress concentration at the point of contact, and therefore give higher piezoelectric output.^147^ Micro-geometric structures tend to show large deformation under small stress, causing a large change in dipole density, which is conducive to the detection of weak mechanical excitation. Some microstructures can also improve strain transfer by eliminating the large stress concentration between materials with elastic modulus mismatch.^163,166^ In addition, microstructures like buckled structures, serpentine structures, and Kirigami designs can improve stretchability while maintaining stable piezoelectric performance, as comprehensively summarized and discussed in Zhou's article.^143^

**Fig. 13:**
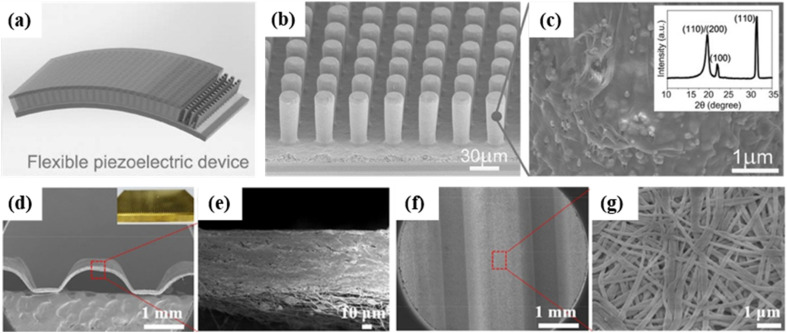
(a) Schematic diagram showing the piezoelectric P(VDF-TrFE)/BaTiO_3_ device. (b) SEM image of the P(VDF-TrFE)/BaTiO_3_ nanocomposite micropillar array. (c) The magnified side-view SEM image of a nanocomposite micropillar. The inset shows the XRD spectrum of the nanocomposite micropillar array. This figure has been reproduced from ref. 164 with permission from John Wiley and Sons, copyright 2017. SEM images of (d) and (e) cross section and (f) and (g) surface morphology of the wave-shaped piezoelectric nanofiber device. The inset shows the copper templates used to fabricate the wave-shaped structures. This figure has been reproduced from ref. 165 with permission from Springer Nature, copyright 2021.

Chen and his colleague developed a high-performance flexible nanogenerator using a piezoelectrically enhanced nanocomposite micropillar array of P(VDF-TrFE)/BaTiO_3_*via* a facile, reliable and scalable nanoimprinting process (Fig. 13a).^164^Fig. 13b and c show the SEM images of imprinted vertically aligned micropillar arrays, and it is clearly observed that BaTiO_3_ NPs were dispersed among the nanocomposite micropillars uniformly. The prepared device produced a piezoelectric voltage that was 7.3 times higher than that of the pristine P(VDF-TrFE) flat film. The superior performance could be partially attributed to the improved mechanical flexibility of the micropillar array under compression. In 2021, Xu *et al.* patterned electrospun PVDF nanofibers by embossing templating to form a wave-shaped 3D structure.^165^ The cross-sectional view and surface morphology of the wave-shaped structure are depicted in Fig. 13d and f, respectively. The embossing process yielded a compact membrane structure with reduced porosity (Fig. 13e), although its surface remained covered with disorderly PVDF nanofibers (Fig. 13g). Both theoretical and experimental results showed that because of the synergistic piezoelectric effects of both *d*_33_ and *d*_31_ modes, the wavy structure enhanced electromechanical coupling, resulting in better longitudinal and transverse piezoelectric performance. This 3D device could effectively distinguish an acoustic frequency difference of at least 0.1 Hz, enabling frequency spectrum analyses of various acoustic sources from human and animals.

### 4.3 Hierarchical structures

A hierarchical structure involves the incorporation of specific structures to optimize the overall performance. The most commonly employed hierarchical structures can be broadly classified into two types: one is composed of multiple individual micro-geometric structures known as combined hierarchical structures in the subsequent section, while the other consists of multiple layers of different materials referred to as multilayer hierarchical structures. In comparison to other simple structures, hierarchical structures consistently exhibit great potential for achieving superior piezoelectric performance, mechanical characteristics, detection sensitivity and durability. It is evident from many existing studies that hierarchical structures significantly enhance the design versatility of diverse piezoelectric materials, spanning from the nanoscale to the micron scale. Typically, the interlocking structures play a vital role in hierarchical structures.^28,142,157,167,168^ This kind of design allows adjacent microstructures to make sufficient contact and support each other well, which can not only cause stress concentration and then effectively improve the response performance, but also prevent structural damage and enhance the stability and durability of sensors.

**Fig. 14:**
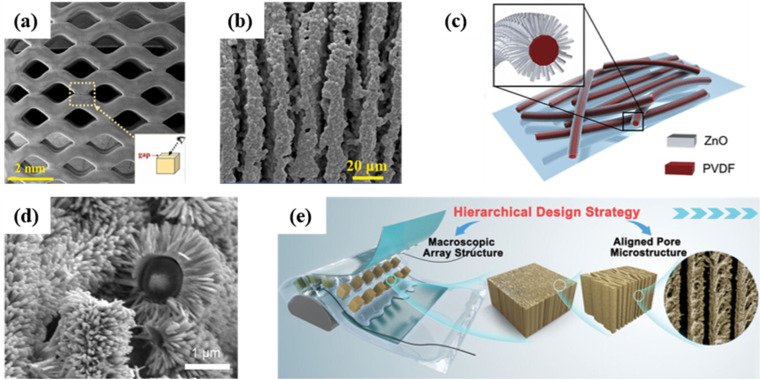
(a) SEM image of millimeter-scale macropores. (b) SEM image of micrometer-scale micropores. This figure has been reproduced from ref. 29 with permission from Elsevier, copyright 2024. (c) Schematic diagram of core–shell PVDF/ZnO nanofibers. (d) SEM image of the interlocked PVDF/ZnO nanofibers. This figure has been reproduced from ref. 28 with permission from Elsevier, copyright 2020. (e) Schematic diagram of the hierarchical design strategy for fabricating aligned porous PZT piezoceramic arrays. This figure has been reproduced from ref. 152 with permission from John Wiley and Sons, copyright 2023.

Combined hierarchical structures have been widely studied in recent years, such as multiscale porous hierarchical structures,^29^ interlocking micro-geometric structures^28^ and porous structures with a geometric structure.^98,150,152^ Recently, Ye's group reported the fabrication of PZT piezoelectric ceramic scaffolds with sophisticated hierarchical porous structures by simultaneous direct ink writing and freeze casting.^29^ The printed scaffolds possessed a combination of millimeter-scale macropores formed by the extrusion process and micrometer-scale micropores formed by the simultaneous freeze casting (Fig. 14a and b). The appropriate structure design effectively improved the piezoelectric response, with a sensitivity of 8.98 V kPa^−1^ and a high output voltage of 191 V under the application of 1 N external force. In 2020, Yang and his colleagues proposed a 3D hierarchically interlocked PVDF/ZnO nanofiber-based piezoelectric sensor by epitaxially growing ZnO nanorods (NRs) on the surface of electrospun PVDF nanofibers (Fig. 14c and d).^28^ The hierarchically interlocked ZnO NRs enabled significant deformation, leading to a stronger piezoelectric potential. The prepared sensor exhibited greatly improved sensitivities that were 6 times and 41 times greater than those of pure PVDF nanofibers, respectively, in pressing and bending modes. In 2023, Xu *et al.* presented a hierarchical design strategy for forming porous piezoceramics with an optimized microstructure, arranged into an ordered macroscopic array structure (Fig. 14e).^152^ The designed device significantly overcame the inherent brittleness and low durability of piezoelectric ceramics, while providing excellent piezoelectric properties with a high open circuit voltage of 618 V, a high short circuit current of 188 μA and an ultrahigh power density of 19.1 mW cm^−2^.

**Fig. 15:**
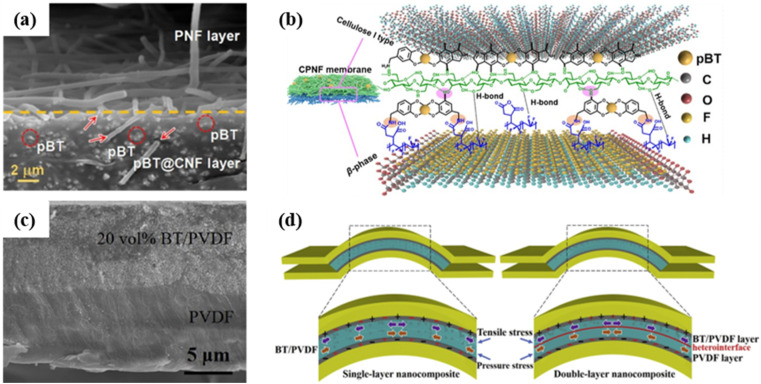
(a) SEM image of the cross-section of CPNF membranes with pBT concentrations of 20.0 wt%. (b) Schematic diagram of the primary intermolecular interactions within the layered CPNF membranes. This figure has been reproduced from ref. 168 with permission from Elsevier, copyright 2022. (c) SEM image of the cross section of nanocomposite films of 20 vol% BaTiO_3_/PVDF suspension. (d) Schematic diagram of the stress and related inductive charges distribution in single-layer and double-layer nanocomposite films. This figure has been reproduced from ref. 170 with permission from Elsevier, copyright 2018.

Compared with other structures, multilayer hierarchical structures offer the advantage of enhanced charge accumulation under identical external forces, thereby improving the sensitivity of piezoelectric sensors.^156,168–170^ However, it is not necessarily advantageous to incorporate more layers in the structure. Ensuring mechanical deformation stability and preventing detachment after long-term use is a crucial consideration. Therefore, selecting an appropriate synthesis method is essential, and the interactions between different layer materials should also be taken into account. The appropriate synthesis method can not only ensure stability but also compensate for defects in different layers, promoting stress transfer.^170^ Wang *et al.* developed piezoelectric nanogenerators with superior piezoelectricity and mechanical robustness by integrating cotton cellulose nanofibers (CNFs) and maleic-anhydride-grafted polyvinylidene fluoride nanofibers (PNFs).^168^ Strong interlayer interactions including chemical and hydrogen bonds were responsible for the mechanical strength and durability of the layer-structured membranes. BaTiO_3_ nanoparticles surface-modified with PDA (pBT), chemically loaded onto the surfaces of CNFs, acted as an interlayer bridge to covalently bind the hydrophilic CNF and hydrophobic PNF layers (Fig. 15a and b). And then the synergetic contributions of these three components greatly improved the piezoelectric outputs. In 2018, Hu's group reported another principle of improving piezoelectric performance through a heterostructure of double-layer (DL), which enhanced the electric capacity of the prepared films.^170^ The DL film consisted of one half made of a high-content BaTiO_3_ nanoparticle layer and another half made of a neat polymer layer created *via* solution spin coating (Fig. 15c). In addition to the interface between the film and the electrodes, the DL structure allowed for increased storage of inductive charges at the additional interfaces between the BaTiO_3_/PVDF layer and neat PVDF layer compared with the single-layer (SL) structure (Fig. 15d). These accumulated inductive charges formed dipoles, thereby inducing enhanced polarization and amplifying the overall piezoelectric response. Moreover, the DL structure was more conducive to piezoelectric output during bending. In a uniform SL film, the opposite induced charges generated by the tensile stress on the upper half and compressive stress on the lower half might cancel out each other. However, the counteracting effect in the DL structure was weak due to different polarization modes and dipole orientations in two different layers, and a greater net piezoelectric response could be induced.

## 5 Applications of wearable piezoelectric sensors

**Fig. 16:**
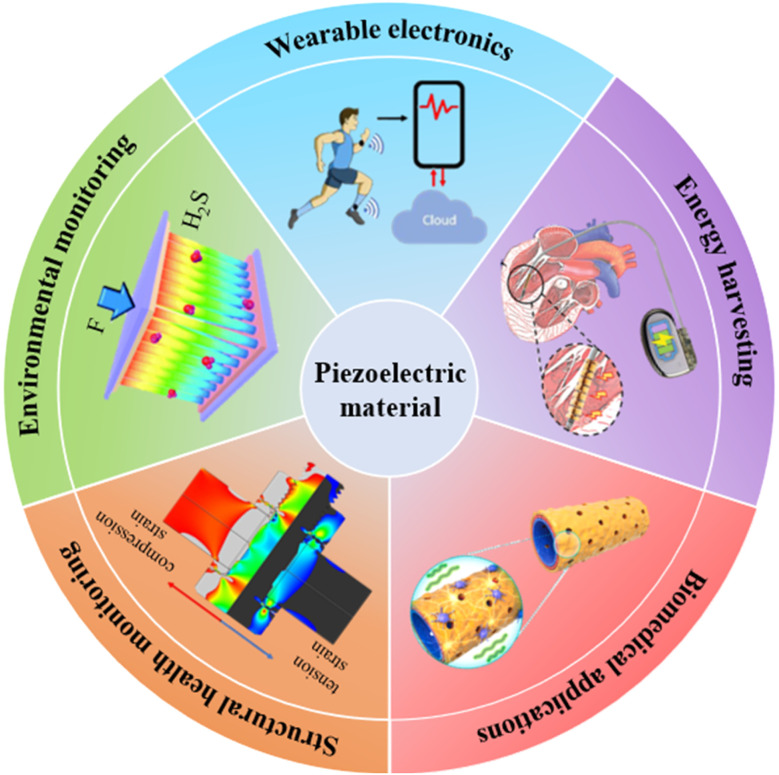
Schematic diagram showing diverse applications of piezoelectric materials with some examples. A wireless real time health monitoring system for wearable electronics. This figure has been reproduced from ref. 34 with permission from Elsevier, copyright 2023. An implantable cardiac piezoelectric composite patch for energy harvesting. This figure has been reproduced from ref. 182 with permission from John Wiley and Sons, copyright 2021. A piezoelectric scaffold conducive to neuronal repair *in vivo* for biomedical applications. This figure has been reproduced from ref. 183 with permission from Elsevier, copyright 2021. A piezoelectric wafer active sensor for structural health monitoring of bolts. This figure has been reproduced from ref. 184 with permission from Springer Nature, copyright 2024. A piezoelectric sensor to detect H_2_S selectively at room temperature for environmental monitoring. This figure has been reproduced from ref. 185 with permission from Royal Society of Chemistry, copyright 2015.

Piezoelectric materials have attracted attention in multiple fields, with a wide range of applications including energy harvesting,^38,171,172^ biomedical applications,^173–175^ structural health monitoring,^176–178^ environmental monitoring^179–181^ and wearable electronics. Fig. 16 briefly summarizes these applications. Piezoelectric energy harvesting is superior to electromagnetic and triboelectric methods due to its high energy conversion performance and high piezoelectric sensitivity. Additionally, polymer materials with significant biodegradability and biocompatibility greatly expand the application range as implantable nanogenerators. Biomedical applications encompass wound healing and tissue engineering. Piezoelectric wound dressings can deliver continuous electrical stimulation to facilitate cell proliferation, differentiation and migration for enhanced wound healing. Given the inherent piezoelectricity observed in several human tissues such as bone, nerve and muscle tissues, organic piezoelectric materials can serve as tissue stimulants and scaffolds to facilitate tissue regeneration. In structural health monitoring, advanced piezoelectric sensors provide electrical responses to stress, allowing for the real-time assessment of the condition, integrity and remaining lifespan of structures. Environmental monitoring utilizes piezoelectric sensors to measure humidity, temperature and detect harmful gases, enabling continuous monitoring to optimize environmental conditions and address issues promptly.

Here we delve into the application of piezoelectric sensors in wearable electronics, with a specific focus on health monitoring and human–machine interaction (HMI). Wearable health monitoring sensors have evolved from simple wrist-worn fitness trackers to multifunctional sensors, which can not only detect human movements such as muscle contractions,^186,187^ joint motions^187^ and walking postures,^88,188^ but also monitor physiological signals including the heart rate,^164,189^ breathing,^190,191^ blood pressure^32,192^ and sleep patterns^193,194^ in real-time. Smart piezoelectric tactile and force sensors also have great potential in the HMI field as the basic and indispensable device for information interaction. With the development of material and structural design, sensitivity, response time, wearing fitness and capability of multi-dimensional tactile sensing have been greatly enhanced. In addition, surface adhesion, breathability, biocompatibility and long-term reliability are also essential elements for wearable sensors due to their long term intimate contact with human skin. Reducing the thickness of electronic devices is a common method to mitigate the intrinsic mechanical mismatch between electronics and human skin. Sensors are always made in the form of ultrathin films,^193^ electronic skins,^195,196^ e-tattoos^197,198^ and patches.^199,200^ Encapsulating devices with biomaterials or using piezoelectric materials with intrinsic biocompatibility can effectively avoid the risk of skin damage.

### 5.1 Health monitoring

Wearable piezoelectric sensors enable non-invasive monitoring of diverse human physical and physiological parameters, which play an increasingly vital role in the future healthcare system with the rapid development and extensive application of information technology. They have shown promising applications in disease prevention, diagnosis and treatment, making the process of treatment more convenient, more timely and easier to manage. Currently, piezoelectric sensors can be utilized for long-term monitoring and timely intervention in cardiovascular diseases (CVD), greatly reducing the mortality rate associated with these diseases.^201,202^ Compared with another commonly used optical method of photoplethysmography, piezoelectric sensors have the advantage of providing additional information for diagnosing CVD, directly reflecting accurate arterial pulse waveforms. Piezoelectric sensors can monitor physiological parameters related to atherosclerosis screening, thereby detecting diseases at an early stage and reducing fatal vascular events.^203^ The vibroarthrography method based on piezoelectric acoustic sensors is recognized as an emerging tool for the detection of knee osteoarthritis.^204^ This method can assess the Kellgren and Lawrence grades of osteoarthritis by analyzing the vibration and sound frequency pattern. It is completely non-invasive, cost-efficient and capable of integrating a real-time examination in the dynamic mode of operation.

**Fig. 17:**
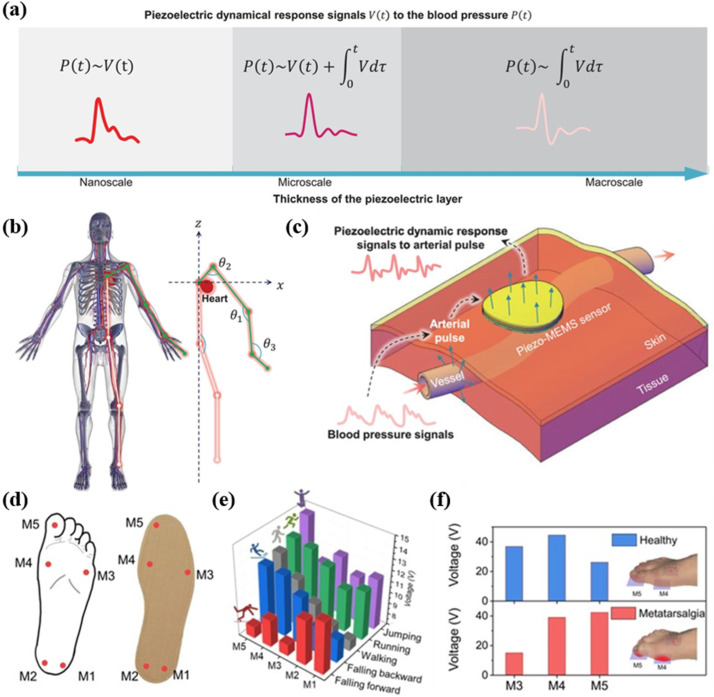
(a) Piezoelectric responses to blood pressure, related to piezoelectric layer thickness. (b) Schematic diagram of the human arterial vessel system and the pulse transmission damping affected by the joint bent angle *θ*. (c) Illustration of the piezoelectric dynamic response to arterial pulse from the arterial vessels to the skin. This figure has been reproduced from ref. 208 with permission from John Wiley and Sons, copyright 2022. (d) Schematic diagram of a smart insole integrated with five PT sensors on five different sites to form a foot sensor network. Signals profiles for (e) gait monitoring and (f) Metatarsalgia prognosis. This figure has been reproduced from ref. 88 with permission from Springer Nature, copyright 2022.

Over the past few years, piezoelectric sensors for wearable continuous blood pressure monitoring have been widely investigated,^205–207^ effectively reducing patients' suffering and infection risk compared to implanting invasive pressure sensors in the center of arteries. Yi and his colleagues successfully resolved the controversy over the arterial pulse wave piezoelectric response, revealing three correlations to relate the piezoelectric response to blood pressure: *via* integration, *via* transition correction and *via* direct correlation (Fig. 17a).^208^ They also found that the motion-artifact due to the posture specificity of the obtained raw arterial pulse waves could be eliminated using arterial-pulse piezoelectric responses (Fig. 17b and c). The used wireless monitoring system with just a single piezoelectric sensor showed greater portability and high prediction accuracy of arterial-pulse identification for primary prevention and daily control of hypertension. Min *et al.* reported a wearable piezoelectric sensor for continuous non-invasive arterial pressure monitoring, solving the problem of the absence of an accurate transfer function to convert the sensor signals into blood-pressure values.^32^ The piezoelectric sensor was able to precisely define the shape of blood pulse due to its high sensitivity (0.062 kPa^−1^), fast response time (23 ms) and conformal contact between the ultrathin flexible sensor and rugged skin. Li *et al.* reported a thin, soft, miniaturized system for continuous arterial blood pressure monitoring, by integrating a sensor array, an active pressure adaptation system and a signal sampling and processing module into a thin and soft wristband.^192^ Benchtop studies, structural design, theoretical simulation and initial trials involving 87 volunteers demonstrated the feasibility and practical utility of the system. This work showed great advantages for addressing the issues related to system integration, interfacial performance and blood pressure estimation model.

Real time monitoring of human movements can provide detailed information about the types and conditions of movements, which greatly contributes to sports injury prevention^88,209,210^ and the effectiveness and accuracy of clinical diagnosis.^188,211^ Su *et al.* fabricated the Ti_3_C_2_T_*x*_/Sm-PMN-PT/PVDF composite-based soft piezoelectric textile sensors *via* electrospinning.^88^ As shown in Fig. 17d, the sensors were attached onto five different positions of a conventional insole to form a foot sensor network for detecting the stress distribution of the foot spontaneously. The synthesized devices could not only accurately recognize and distinguish various gait patterns of walking, running, jumping, falling forward and falling backward, but also show ability to identify walking habits and predict Metatarsalgia (Fig. 17e and f). Gao and his colleagues proposed a facile and scalable strategy for fabricating textured piezoelectric composites for high-precision flexible sensing and human motion monitoring.^209^ A flexible sports health monitoring system based on the piezoelectric composites was developed, which could seamlessly fit vulnerable joints like a band-aid, including ankle, wrist and knee joints. The system demonstrated promising potential for assisting rehabilitation in sprained patients by providing real-time warning of risky actions, preventing chronic sports injuries among badminton fans by rectifying wrong exertion or posture. Xie *et al.* developed a wearable multisource gait monitoring system for the gait analysis and objective evaluation of Parkinson's diseases (PD), by integrating force sensitive sensors, piezoelectric sensors and inertial measurement units.^188^ By utilizing the designed system to collect multisource gait data from PD patients and healthy controls, features for quantitative analysis of gait abnormalities were extracted. The results showed that the duration of gait phases, dynamic deformation and postural angles all had the diagnostic capability for PD.

### 5.2 Human–machine interaction

Human–machine interaction refers to the communication and interaction between a human and a machine *via* a user interface. HMI is gaining increasing attention due to its broad application prospect in various fields such as robot manipulation, smart prosthetics and entertainment. Advanced sensors play a crucial role in HMI, perceiving external stimuli and converting various detected physical parameters into input signals transmitted to the machine system.^212–214^ Piezoelectric sensors perform well in key evaluation indicators such as sensitivity, stretchability, resolution, stability, size and biocompatibility. Moreover, they possess the advantages of high power density and superior energy conversion efficiency, and their inherent self-powering characteristics give them a significant edge in terms of energy conservation. Piezoelectric sensors can also meet the demands for high integration and miniaturization, and can be fabricated into ultra-thin structures, ensuring exceptional performance while guaranteeing comfort and portability. The main trend of current technology is to endow sensors with intelligence to enhance their information processing efficiency.^142,215^ This allows HMI to provide the same required function just with a minimal number of sensors.

**Fig. 18:**
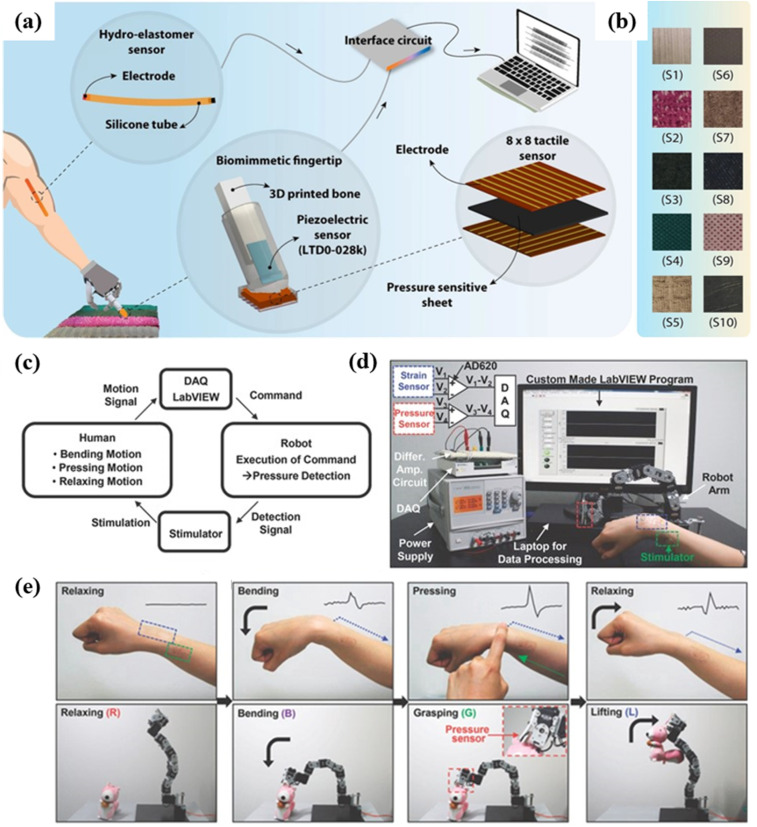
(a) Schematic diagram showing the proposed multi-channel neuromorphic system. (b) Ten different naturalistic fine textures. This figure has been reproduced from ref. 221 with permission from Springer Nature, copyright 2022. (c) A flow chart and (d) the corresponding experimental setup for HMI demonstration. (e) The transparent motion sensor (blue dotted box) and the electrotactile stimulator (green dotted box) were attached to the top side of the wrist and the lateral side of the forearm, respectively. The robot arm remained still if no motion was detected. The bending, pressing and relaxing of the wrist caused the robot arm to bend, grasp and lift, respectively. This figure has been reproduced from ref. 33 with permission from John Wiley and Sons, copyright 2014.

Due to their excellent performance, HMIs based on piezoelectric sensors show promising potential in smart prosthetics,^216–218^ which allow users to interact with the surrounding environment and regain the normal activities. Piezoelectric sensors provide sensory feedback,^219,220^ particularly from touch or physical contact, enabling prosthetics to simultaneously perceive and distinguish multiple tactile stimuli such as pressure, vibration and pain. Rostamian *et al.* fabricated a soft biomimetic fingertip including an 8 × 8 array of tactile sensors and a piezoelectric sensor to mimic Merkel, Meissner and Pacinian mechanoreceptors in glabrous skin.^221^ Then a hydro-elastomer sensor providing proprioceptive feedback, replicating muscle spindles, was integrated with the fingertip (Fig. 18a). The prosthetic hand could discriminate all 10 fine naturalistic textures with 6 different scanning speeds (Fig. 18b). The integration of multiple sensory feedback systems in prosthetic hands was conducive to dealing with the external environment, slip detection and fine motor activities. Jasni and his colleagues studied a piezoelectric-based in-socket sensory system for detecting the gait phases and gait phases' transitions in transfemoral amputees.^222^ An array of piezoelectric sensors was used to capture the force profile of the stump while it was inside the prosthetic socket and performing gait. The information extracted from the gait analysis served as input to the controller of the micro-processor controlled prosthetic leg and eventually helped to decide the next action to be performed. From the study results, it was observed that the in-socket sensory system was able to detect the difference in the walking type or speed by the amputee, efficient for the micro-processor controlled prosthetic leg.

In HMI, flexible sensors are also considered as the most important components in the application of robot manipulation.^33,223,224^ The signals received by piezoelectric sensors are processed, recognized and then translated into a set of predefined commands for remote control and operation of smart devices. Lim and his colleagues developed a HMI system composed of wearable sensors and stimulators that could demonstrate a robot control and transfer feedback signals to the operator (Fig. 18c and d).^33^ The ultrathin and serpentine design endowed the entire system with excellent stretchability, enabling conformal integration with human skin and collecting signals with minimal motion artifacts. The HMI system enabled elaborate interactive robot control: when piezoelectric sensors detected human movements like wrist relaxation, bending and pressing, the robotic arm would respond with corresponding bending, gripping and lifting actions based on pre-designated commands in the software (Fig. 18e). Apart from the mostly extensively studied remote gesture control, Bouyam *et al.* also proposed the HMI system for wheelchair control for quadriplegic patients.^224^ Six piezoelectric sensors were employed to acquire facial muscle signals during winking and tongue movements. With the proposed algorithm, the command translation patterns could reach more than 95% of the average classification accuracy. The results demonstrated that integrating tongue actions and winking yielded high efficiency similar to joystick control.

## 6 Conclusion and perspectives

**Fig. 19:**
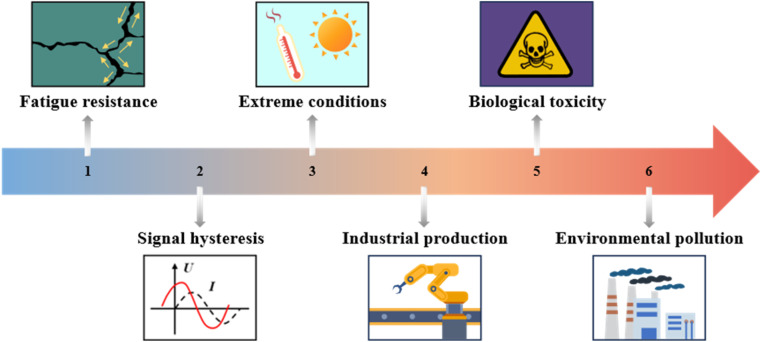
Perspective for future research on wearable piezoelectric sensors.

In this review, wearable piezoelectric sensors are comprehensively summarized and discussed focusing on their recent progress in material design, engineering strategy and wearable applications. In the past few years, flexible piezoelectric sensors have witnessed greatly enhanced performance through the development of all sorts of novel materials and structures, improving sensitivity, stretchability, detection range, durability and self-powered ability. For instance, PVDF and P(VDF-TrFE), most commonly studied piezoelectric polymer materials, can be engineered to increase the β phase proportion to improve the piezoelectric properties. On the other hand, electrospinning technology has rapidly evolved from single fluid processes to coaxial, triaxial, parallel and three-layer parallel processes,^225^ making it a promising method for preparing complicated nanostructures. Piezoelectric hydrogels, although still in their infancy, have exhibited specific advantages for wearable applications apart from their high performance, such as self-healing, anti-freezing and adhesive capabilities. The designed microstructures, whether porous structures or micro-geometric structures or hierarchical structures, have certainly offered practical and feasible methods for developing better-performing piezoelectric sensors. Moreover, a detailed discussion on important applications in the areas of health monitoring and human–machine interaction is presented. The innovations in artificial intelligence, big data and IoT enormously promote smart applications of flexible piezoelectric sensors. In spite of encouraging progress made to date, there exist several hurdles to overcome in future research, as described in Fig. 19.

First, the durability and fatigue resistance of piezoelectric sensors affect signal integrity and determine their service life under practical conditions. Continuous, repetitive and intense external forces can lead to damage or even disappearance of nanostructures, resulting in decreased sensitivity. Incorporation of heterojunctions is a potential strategy to enhance phase interfaces inside the material structure. Second, piezoelectric hydrogels fail to return to their original state under extreme strain, which leads to considerable signal hysteresis. Hydrogels also face the problems of water loss and ion leakage, which can lead to the loss of electrical conductivity and mechanical properties, and then instability or even deterioration of sensing performance. Thus, more attention should be paid to structure design, composite phase and interface regulation of piezoelectric hydrogels to address signal hysteresis, water loss and ion leakage issues. Third, piezoelectric sensors are negatively sensitive to environmental conditions, such as temperature, humidity and water. Research studies have shown that the piezoelectric effect of materials tends to increase with decreasing temperature.^226^ Therefore, more advanced methods for hermetic packaging and stabilization are desired to address this issue. Fourth, another urgent problem to be solved is the large-scale industrial production of high-performance piezoelectric sensors, and most research studies focus on laboratory-scale manufacturing techniques, far from the requirements of commercialization. For the most commonly used fabrication methods, both 3D printing and electrospinning require specialized equipment with high costs. The template method is relatively simple, but it requires consideration of complex processes and the possibility of breakage during peeling. Notably, 3D printing is an emerging industrial technology that has potential for large-scale fabrication, but it has not yet been standardized to the same level as traditional manufacturing. Fifth, it is an important challenge to eliminate skin damage induced by biotoxicity of sensing materials especially nanomaterials inside wearable sensors. A biological toxicity assessment system should be established for sensor materials and devices, and strict material safety standards need to be taken into account during device preparation. Lastly, per- and polyfluoroalkyl substances (PFAS) have emerged as a persistent environmental pollutant, demanding urgent attention.^227^ Although PVDF and its derivatives are chemically stable and unlikely to harm human health, they may still pose PFAS hazards during production and disposal. More environmentally friendly processes should be used for PVDF preparation, such as the use of non-toxic solvents and solvent-free strategies. Reusing and recycling are viable waste management options. PVDF exhibits good sustainability and can be reused up to five times without significant loss in physical performance.^228^ However, the presence of fillers and other substances often contaminates PVDF, making recycling a challenge. The ultimate treatment of waste through incineration or landfill inevitably results in environmental pollution, making it crucial yet challenging to develop biodegradable alternatives.

## Data availability

No primary research results, software or code have been included and no new data were generated or analysed as part of this review.

## Author contributions

Yanyu Chen: visualization, investigation, writing – original draft. Xiaohong Zhang: formal analysis, validation. Chao Lu: conceptualization, supervision, project administration, funding acquisition, writing – review & editing.

## Conflicts of interest

The authors declare no conflict of interest.
